# Impact of Angiotensin-Converting Enzyme Inhibitors and Angiotensin Receptor Blockers on the Inflammatory Response and Viral Clearance in COVID-19 Patients

**DOI:** 10.3389/fcvm.2021.710946

**Published:** 2021-08-19

**Authors:** Linna Huang, Ziying Chen, Lan Ni, Lei Chen, Changzhi Zhou, Chang Gao, Xiaojing Wu, Lin Hua, Xu Huang, Xiaoyang Cui, Ye Tian, Zeyu Zhang, Qingyuan Zhan

**Affiliations:** ^1^Center for Respiratory Diseases, China-Japan Friendship Hospital, Beijing, China; ^2^Department of Pulmonary and Critical Care Medicine, China-Japan Friendship Hospital, Beijing, China; ^3^National Clinical Research Center for Respiratory Diseases, Beijing, China; ^4^Peking University Health Science Center, Beijing, China; ^5^Department of Pulmonary and Critical Care Medicine, Zhongnan Hospital of Wuhan University, Wuhan, China; ^6^Department of Pulmonary and Critical Care Medicine, Tongji Hospital, Tongji Medical College, Huazhong University of Science and Technology, Wuhan, China; ^7^Department of Pulmonary and Critical Care Medicine, The Central Hospital of Wuhan, Wuhan, China; ^8^Department of Critical Care Medicine, The First Affiliated Hospital of Soochow University, Suzhou, China; ^9^School of Biomedical Engineering, Capital Medical University, Beijing, China

**Keywords:** ACE inhibitor, ARB, inflammatory response, viral clearance, COVID-19

## Abstract

**Objectives:** To evaluate the impact of angiotensin-converting enzyme inhibitors (ACEIs) or angiotensin receptor blockers (ARBs) on the inflammatory response and viral clearance in coronavirus disease 2019 (COVID-19) patients.

**Methods:** We included 229 patients with confirmed COVID-19 in a multicenter, retrospective cohort study. Propensity score matching at a ratio of 1:3 was introduced to eliminate potential confounders. Patients were assigned to the ACEI/ARB group (*n* = 38) or control group (*n* = 114) according to whether they were current users of medication.

**Results:** Compared to the control group, patients in the ACEI/ARB group had lower levels of plasma IL-1β [(6.20 ± 0.38) vs. (9.30 ± 0.31) pg/ml, *P* = 0.020], IL-6 [(31.86 ± 4.07) vs. (48.47 ± 3.11) pg/ml, *P* = 0.041], IL-8 [(34.66 ± 1.90) vs. (47.93 ± 1.21) pg/ml, *P* = 0.027], and TNF-α [(6.11 ± 0.88) vs. (12.73 ± 0.26) pg/ml, *P* < 0.01]. Current users of ACEIs/ARBs seemed to have a higher rate of vasoconstrictive agents (20 vs. 6%, *P* < 0.01) than the control group. Decreased lymphocyte counts [(0.76 ± 0.31) vs. (1.01 ± 0.45)^*^10^9^/L, *P* = 0.027] and elevated plasma levels of IL-10 [(9.91 ± 0.42) vs. (5.26 ± 0.21) pg/ml, *P* = 0.012] were also important discoveries in the ACEI/ARB group. Patients in the ACEI/ARB group had a prolonged duration of viral shedding [(24 ± 5) vs. (18 ± 5) days, *P* = 0.034] and increased length of hospitalization [(24 ± 11) vs. (15 ± 7) days, *P* < 0.01]. These trends were similar in patients with hypertension.

**Conclusions:** Our findings did not provide evidence for a significant association between ACEI/ARB treatment and COVID-19 mortality. ACEIs/ARBs might decrease proinflammatory cytokines, but antiviral treatment should be enforced, and hemodynamics should be monitored closely. Since the limited influence on the ACEI/ARB treatment, they should not be withdrawn if there was no formal contraindication.

## Introduction

Up to March 31, 2020, the total number of patients with coronavirus disease 2019 has risen sharply to nearly 700,000 globally, with a mortality rate of nearly 5%. Meanwhile, this epidemic seems to be spreading at an exponential rate and has become an urgent public health emergency of international concern.

Several large retrospective studies have revealed that pre-existing cardiovascular disease and diabetes were the most frequent comorbidities of coronavirus disease 2019 (COVID-19) patients (1–3); these patients even had a higher risk of mortality (4, 5) than those with underlying respiratory disease. Angiotensin-converting enzyme inhibitors (ACEIs) and angiotensin receptor blockers (ARBs) are widely prescribed for these patients. ACEIs/ARBs have an impact on the renin-angiotensin system (RAS) and are postulated to attenuate pulmonary and systemic inflammatory responses, reducing the severity and mortality of viral pneumonia-related acute respiratory distress syndrome (6–8), ultimately by angiotensin-converting enzyme 2 (ACE2) upregulation through the ACE2-Ang-(1-7)-Mas axis (9).

The molecular biology of severe acute respiratory syndrome coronavirus 2 (SARS-CoV-2) is well-established, as it appears to bind to its target cells through ACE2, which is expressed by epithelial cells of the lung, to enable it to infect host cells (10, 11). The expression of ACE2 is substantially increased in patients who are treated with ACE inhibitors and ARBs (12), which promotes SARS-CoV-2 entry into the body, increasing the risk of developing COVID-19 (13, 14).

The controversial pathogenesis as well as the mixed results of several clinical studies (15, 16) of pneumonia with other pathogens made it difficult for physicians to determine whether the use of ACE inhibitors or ARBs should be terminated in patients with COVID-19.

To date, the actual impact of ACE inhibitor and ARB prescriptions on COVID-19 patients has not been assessed in current studies. Therefore, we aimed to evaluate the clinical manifestations and outcomes, especially inflammatory responses and viral clearance, by a multicenter, retrospective cohort study.

## Materials and Methods

### Study Design and Population

We retrospectively included patients with microbiologically confirmed cases of COVID-19 according to the World Health Organization (WHO) (17) and official Chinese guidelines (18) in a multicenter retrospective cohort study performed at three tertiary hospitals in Wuhan, Hubei Province, China (Tongji Hospital, Tongji Medical College, Huazhong University of Science and Technology; Zhongnan Hospital of Wuhan University; and the Central Hospital of Wuhan) from February 15, 2020 to March 25, 2020. Patients included in our study were all assessed for eligibility on the basis of positive SARS-CoV-2 nucleic acid testing results by reverse transcription-polymerase chain reaction (RT-PCR) with nasopharyngeal swab samples. However, it was not possible to determine whether the patients had pneumonia, as not all were available for CT scans.

#### Exclusion Criteria

(1) Patients younger than 18 years old.
(2) Patients still hospitalized at the end of the study.

All patients were treated according to the standard protocols for antiviral, antibiotic, glucocorticoid, and Chinese medicine treatments.

The ethics committee of China-Japan Friendship Hospital approved this study (2020-21-K16). Written informed consent was waived due to the rapid emergence of this infectious disease.

#### Group Division

We divided the patients into two groups. The ACEI/ARB group included patients who were current users of ACE inhibitors or ARB medication, while non-current users were included as the control group. Patients in the ACEI/ARB group were further divided into subgroups of a continued medication group and a terminated medication group according to the application of ACE inhibitors or ARBs during hospitalization.

### Data Collection and Analysis

We collected data on the following parameters from the hospital electronic medical record systems, nursing records, laboratory examination systems, and radiological examinations and obtained standardized data collection forms: demographic characteristics, comorbidities, medication history within 1 month, symptoms at admission, laboratory finding changes from day 1 to day 14, radiological manifestations, treatment during hospitalization and outcome data that contained the rate of in-hospital death and progression, the duration of viral shedding, the length of hospital stay and the time from onset to death or discharge. The primary outcome was mortality at discharge, while the secondary outcomes we observed included the duration of hospital stay, the duration of viral shedding and the differences in inflammatory cytokines.

Patients with cardiovascular disease and diabetes are often taking a combination of medications with statins (19) and oral hypoglycemic agents, especially thiazolidinediones, which have been reported to have an impact on the level of ACE2 by several studies (14, 20). To further control for potential confounders, data on the use of statins, thiazolidinediones and other antihypertensive agents (α receptor blocking agents, β receptor blocking agents, calcium channel blockers and diuretics) prior to admission in each group were calculated within 90 days (6).

Two researchers also independently reviewed the data collection forms to double check the data collected. Any missing or uncertain records of the epidemiological, medication and symptom data were collected and clarified through direct communication with patients and their families.

We compared the two groups in terms of the above aspects to identify the differences between current users and non-users prior to admission. Then, among the current users of ACEIs/ARBs, an analysis was conducted by comparing the dynamic changes in indicators involved in immune status and inflammatory reactions, as well as the outcomes between patients who continued and terminated medication during hospitalization. As hypertension itself could activate the RAS, patients with hypertension were excluded to avoid potential confounders. A comparison of the immune status, inflammatory reactions and outcomes between the ACEI/ARB and control groups in patients without hypertension was conducted.

### Cytokine and Chemokine Measurement

To evaluate the impact of coronavirus and additional ACE inhibitors or ARBs on the production of cytokines or chemokines in the acute phase of the illness, plasma cytokines and chemokines [interleukin 1β (IL-1β), IL-2R, IL-6, IL-8, IL-10, and tumor necrosis factor α (TNF-α)] were measured using chemiluminescent immunoassays (CLIAs) (CFDA approved) by Siemens IMMULITE 1000 for patients according to the manufacturer's instructions.

### Definitions

Medications classified as ACE inhibitors were benazepril, perindopril and fosinopril, while the ARBs of the included patients were candesartan, irbesartan, valsartan, olmesartan, telmisartan, and losartan.

Patients were considered a current user of medication if they had a supply of medication to last until the date of hospitalization assuming an 80% compliance rate (6, 21). The patients who did not meet the definition were regarded as non-current users. ACE inhibitors or ARBs were considered to be continued if they were given more than 50% of the days during hospitalization (8); otherwise, they were considered to be terminated.

In-hospital progression was defined as a decline in PaO_2_/FiO_2_ of more than 100 mmHg or the need for invasive positive pressure ventilation (IPPV) and/or extracorporeal membrane oxygenation (ECMO) during hospitalization.

The duration of viral shedding was defined as the duration of the SARS-CoV-2 RNA test result becoming negative from positive. All patients were routinely reexamined for SARS-CoV-2 nucleic acid testing every 5 days to assess whether it had turned negative.

Shock was defined according to the interim guidance of the WHO for novel coronavirus (22). Acute kidney injury (AKI) was identified and classified on the basis of the highest serum creatinine level or urine output criteria according to the Kidney Disease Improving Global Outcomes Classification (KDIGO) (22, 23). Respiratory failure, coagulation and liver failure were defined as a Sequential Organ Failure Assessment (SOFA) score greater than or equal to two points.

### Statistical Analysis

Descriptive statistics included proportions for categorical variables and the mean (standard deviation) or median (interquartile range) for continuous variables. Data were unadjusted unless specifically stated otherwise.

#### Processing of Missing Data

When the missing rate of vital variables involved in our study was <15%, we used SAS predictive mean matching imputation to replace missing values within each variable, while the variables were abandoned when the missing rate reached 20%.

#### Processing of the Unbalanced Sample Size: Propensity Score Matching

The propensity score matching (PSM) method was applied at a ratio of 1:3 between the ACEI/ARB group and the control group. The Sequential Organ Failure Assessment (SOFA) score, Charlson's comorbidity index (CCI), and body mass index (BMI) were matched variables in PSM to derive the cohort. The overall balance test was conducted to confirm that the baseline data of the two groups matched successfully.

Proportions were compared using χ^2^ or Fisher's exact tests, and continuous variables were compared using the *t*-test or Wilcoxon rank sum test, as appropriate. Statistical significance was defined as a two-tailed *P*-value of ≤ 0.05. SAS software, version 9.4 (SAS Institute Inc.) was used for all analyses.

## Results

**Figure 1:**
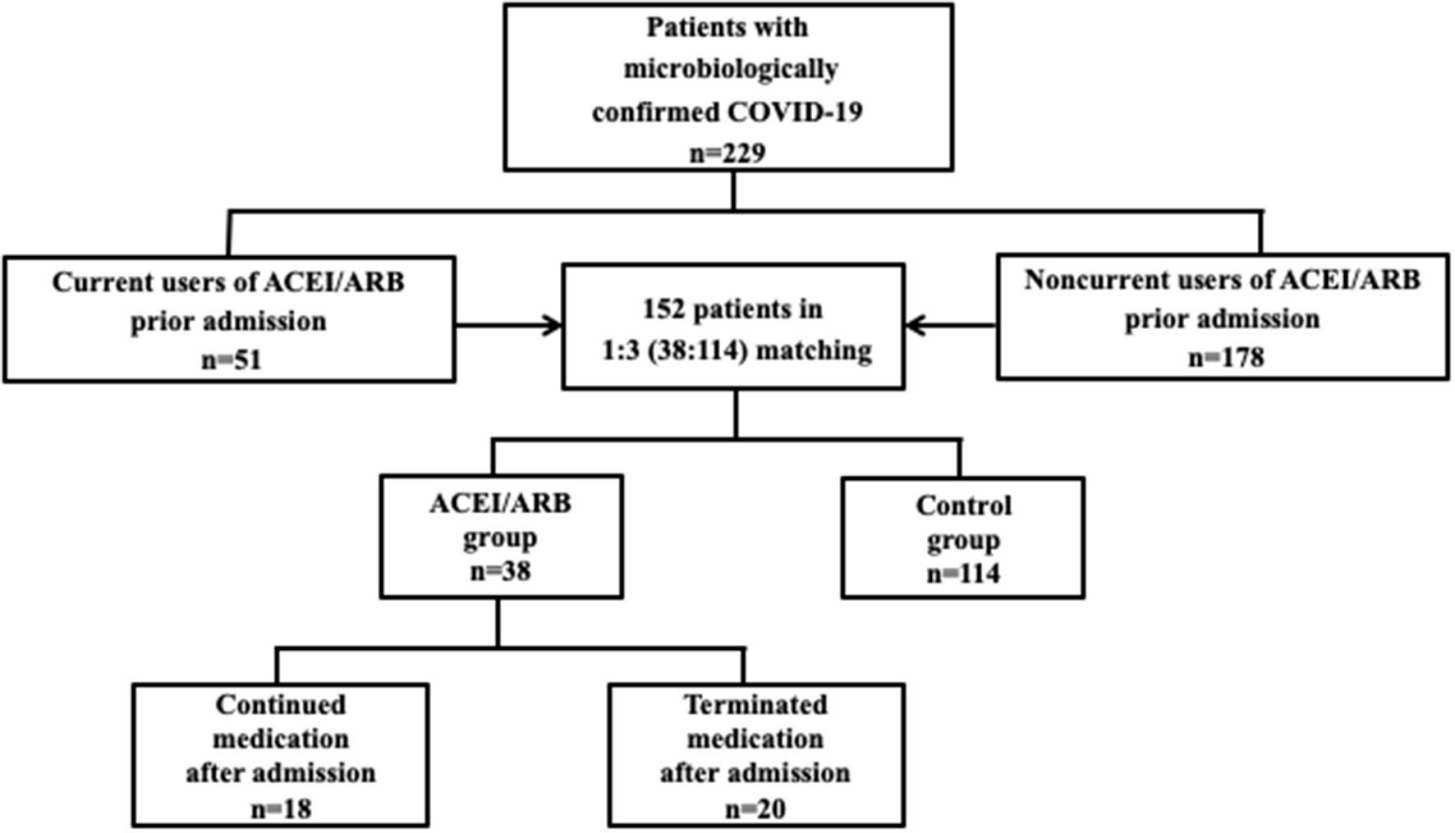
Flowchart. A flowchart illustrated the enrollment of patients in our study. From February 15, 2020 to March 25, 2020, a total of 229 patients with confirmed cases of COVID-19 were admitted; 51 patients were current users of ACEIs/ARBs, while the other 178 patients were non-current users of the medication. The PSM method was applied at a ratio of 1:3 between the ACEI/ARB group (*n* = 38) and the control group (*n* = 114). The SOFA score and CCI were matched variables in PSM to derive the cohort. Among the patients with ACEI/ARB medication, 18 continued medication during hospitalization, while the other 20 terminated medication.

From February 15, 2020 to March 25, 2020, a total of 229 patients with confirmed cases of COVID-19 were admitted; 51 patients were current users of ACEIs/ARBs, while the other 178 patients were non-current users of the medication. The PSM method was applied at a ratio of 1:3 between the ACEI/ARB group (*n* = 38) and the control group (*n* = 114). The SOFA score and CCI were matched variables in PSM to derive the cohort. Thirteen cases in the ACEI/ARB group and 64 cases in the control group were not matched successfully. The overall balance test was with no significant difference between the two groups (*P* = 0.872). Among the patients with ACEI/ARB medication, 18 continued medication during hospitalization, while the other 20 terminated medication (Figure 1). The mean age was 57 ± 12 years, male patients accounted for 52% (*n* = 79), the SOFA score was 1.5 (1–2.3) points, and the CCI was 1 (1–2) prior to admission.

### Comparisons of Baseline Prior Hospitalization Between the ACEI/ARB and Control Groups

The ACEI/ARB group included more patients with hypertension (67 vs. 22%, *P* < 0.01) than the control group. The demographic characteristics, other comorbidities, severity of the condition and possible medication histories might have influenced the ACE2 level but did not differ significantly between the two groups. No significant difference was found between the two groups in time from onset to hospitalization and to COVID-19 diagnosis (Table 1).

**Table 1:** Baseline variables in the two groups prior to admission.

	**All (*n* = 152)**	**ACEI/ARB group (*n* = 38)**	**Control group (*n* = 114)**	***P***
**Age, years, mean** **±** **SD**	57 ± 12	57 ± 11	58 ± 18	0.671
**Gender (men), number (%)**	79 (52%)	19 (51%)	60 (53%)	0.533
**Body mass index, kg/m**^**2**^, **mean±SD**	21.0 ± 6.9	21.1 ± 6.4	21.0 ± 7.0	0.838
**Comorbidities, number (%)**				
Hypertension	55 (36%)	30 (67%)	25 (22%)	<0.001^b^
Diabetes	37 (24%)	10 (27%)	27 (24%)	0.217
Coronary heart disease	17 (11%)	6 (16%)	11 (10%)	0.071
Chronic heart failure	6 (4%)	2 (5%)	4 (4%)	0.622
Underlying lung disease	18 (12%)	7 (18%)	11 (10%)	0.094
Chronic kidney disease	2 (1%)	1 (3%)	1 (1%)	0.512
Chronic liver dysfunction	3 (2%)	0 (0%)	3 (3%)	0.425
Malignancy	3 (2%)	0 (0%)	3 (3%)	0.186
**History of smoking, number (%)**	23 (15%)	8 (21%)	15 (13%)	0.081
**Other medication history within 90 days, number (%)**				
Corticosteroids	0 (0%)	0 (0%)	0 (0%)	1
Immunosuppressants	0 (0%)	0 (0%)	0 (0%)	1
Statins	21 (14%)	6 (16%)	15 (13%)	0.214
Thiazolidinediones	1 (1%)	0 (0%)	1 (1%)	0.996
α receptor blocking agent	4 (3%)	1 (3%)	3 (3%)	0.820
β receptor blocking agent	19 (13%)	5 (13%)	14 (12%)	0.731
CCB	19 (13%)	5 (13%)	14 (12%)	0.731
Diuretics	16 (11%)	4 (11%)	12 (11%)	1
**SOFA Score, points (IQR)**	1.5 (1–2.3)	1.5 (1–2.5)	1.5 (1–2)	0.879
**CCI, points (IQR)**	1 (1–2)	1 (1–2)	1 (1–2)	1
**Treatment before hospital, number (%)**				
Methylprednisolone	10 (7%)	3 (8%)	7 (6%)	0.091
Antibiotic therapy	92 (61%)	22 (58%)	70 (61%)	0.429
Antiviral therapy	102 (67%)	22 (57%)	80 (70%)	0.239
**Time from onset to hospital admission, days, mean** **±** **SD**	10 ± 6	11 ± 3	10 ± 6	0.296
**Time from onset to diagnosis, days, mean** **±** **SD**	7 ± 5	7 ± 5	7 ± 2	0.8

b *P < 0.01; CCB, calcium channel blocker; SOFA, Sequential Organ Failure Assessment; CCI, Charlson's Comorbidity Index (18)*.

### Comparisons of Clinical Symptoms, Laboratory Examinations, and Radiological Manifestations on Admission Between the ACEI/ARB and Control Groups

The symptoms, including fever, cough, hemoptysis, dyspnea, fatigue/myalgia and diarrhea, as well as vital signs, with the exception of systolic blood pressure, were not significantly different between the ACEI/ARB group and the control group. Although systolic blood pressure was lower in the study group (116 ± 14 vs. 124 ± 13 mmHg, *P* = 0.031), it was within the normal range. For laboratory examinations, patients with ACE inhibitor or ARB medication had lower lymphocyte counts [(0.76 ± 0.31) vs. (1.01 ± 0.45) ^*^10^9^/L, *P* = 0.027] than the control group (Table 2).

**Table 2:** Clinical, laboratory findings, and radiological manifestations in the two groups on admission.

	**All** **(***n*** = 152)**	**ACEI/ARB group** **(***n*** = 38)**	**Control group** **(***n*** = 114)**	***P***
**Initial symptoms, number (%)**				
Fever (≥37.3°C)	140 (92%)	35 (92%)	105 (92%)	0.981
Cough	109 (72%)	27 (70%)	82 (72%)	0.866
Productive cough	60 (39%)	16 (42%)	44 (39%)	0.605
Hemoptysis	3 (2%)	1 (3%)	2 (2%)	0.263
Dyspnea	78 (51%)	20 (53%)	58 (51%)	0.432
Fatigue or myalgia	67 (44%)	16 (43%)	51 (45%)	0.619
Diarrhea	46 (30%)	12 (31%)	34 (30%)	0.764
**Initial signs, mean** **±** **SD**				
Highest temperature, °C	38.4 ± 0.7	38.5 ± 1.1	38.3 ± 0.4	0.461
Respiratory rate, breaths/min	23 ± 3	22 ± 3	23 ± 3	0.709
Heart rate, beats/min	96 ± 11	97 ± 8	96 ± 14	0.338
Systolic blood pressure, mmHg	123 ± 10	116 ± 14	124 ± 13	0.031^a^
SpO_2_, %	94 ± 4	93 ± 3	94 ± 4	0.741
FiO_2_, %	40 ± 18	42 ± 15	40 ± 17	0.302
**Laboratory examination, mean** **±** **SD**				
**Blood routine**				
WBC, *10^9^/L	5.94 ± 3.00	6.27 ± 3.21	5.80 ± 2.97	0.085
Neutrophil count, *10^9^/L	4.40 ± 2.99	5.21 ± 3.29	4.39 ± 3.01	0.097
Lymphocytes, *10^9^/L	0.89 ± 0.40	0.76 ± 0.31	1.01 ± 0.45	0.027^a^
**Biochemical examination**				
ALT, U/L	43 ± 4	42 ± 4	43 ± 4	0.747
AST, U/L	40 ± 5	44 ± 4	40 ± 5	0.841
TBIL, mmol/L	11.3 ± 5.2	11.0 ± 5.9	11.4 ± 5.0	0.660
Scr, μmol/L	79.2 ± 2.7	77.5 ± 2.2	80.1 ± 3.6	0.915
LDH, U/L	295 ± 89	301 ± 77	294 ± 91	0.617
TnT, pg/ml	11 ± 1	12 ± 1	11 ± 1	0.770
NT-proBNP, pg/ml	401 ± 55	411 ± 55	397 ± 51	0.528
**Inflammatory factors**				
IL-1β, pg/ml	8.02 ± 0.33	6.20 ± 0.38	9.30 ± 0.31	0.020^a^
IL-2R, U/ml	796.02 ± 27.40	724.25 ± 52.30	807.23 ± 26.21	0.246
IL-6, pg/ml	47.11 ± 3.26	31.86 ± 4.07	48.47 ± 3.11	0.041^a^
IL-8, pg/ml	46.03 ± 1.85	34.66 ± 1.90	47.93 ± 1.21	0.027^a^
IL-10, pg/ml	6.37 ± 0.37	9.91 ± 0.42	5.26 ± 0.21	0.012^b^
TNF-α, pg/ml	11.21 ± 0.44	6.11 ± 0.88	12.73 ± 0.26	<0.001^b^
PCT, ng/ml	0.27 ± 0.07	0.26 ± 0.03	0.29 ± 0.08	0.619
**Coagulation function**				
PT, s	14 ± 3	14 ± 1	14 ± 1	0.995
APTT, s	42 ± 5	44 ± 3	42 ± 5	0.881
D-Dimer, μg/ml	2.19 ± 0.44	2.33 ± 0.47	2.12 ± 0.46	0.448
**Chest CT manifestations, number (%)**				
Bilateral lesion	82 (54%)	19 (49%)	63 (55%)	0.374
GGO	89 (59%)	19 (49%)	70 (61%)	0.310
Consolidation	36 (24%)	11 (29%)	25 (22%)	0.229

a *P < 0.05*;

b *P < 0.01; SpO_2_, saturation of peripheral oxygen; FiO_2_, fraction of inspiration; ALT, alanine aminotransferase; AST, aspartate aminotransferase; TBIL, total bilirubin; Scr, creatinine; LDH; lactate dehydrogenase; TnT, troponin T; NT-proBNP, N-terminal pro-brain natriuretic peptide; IL-1β, interleukin-1β; IL-2R, interleukin-2R; IL-6, interleukin-6; IL-8, interleukin-8; IL-10, interleukin-10; TNF-α, tumor necrosis factor-α; PCT, procalcitonin; PT, prothrombin time; APTT, activated partial thromboplastin time; GGO, ground-glass opacity*.

The first measurements of the inflammatory factors, including IL-1β, IL-2R, IL-6, IL-8, IL-10, and TNFα, were taken within 3 days of admission; while the most (97%, 147/152) were within 24 h. The time from COVID-19 diagnose to measurements was (3 ± 2) days. Besides, as the missing rate reached 12–15%, SAS predictive mean matching imputation was applied to replace missing values in each group. The missing rates of IL-2R, serum ferritin, erythrocyte sedimentation rate (ESR) and C-reactive protein (CRP) were as high as 25–35%; therefore, they were abandoned in the statistical analysis. Patients in the ACEI/ARB group had slightly lower levels of proinflammatory cytokines, including IL-1β [(6.20 ± 0.38) vs. (9.30 ± 0.31) pg/ml, *P* = 0.020], IL-6 [(31.86 ± 4.07) vs. (48.47 ± 3.11) pg/ml, *P* = 0.041], IL-8 [(34.66 ± 1.90) vs. (47.93 ± 1.21) pg/ml, *P* = 0.027], and TNF-α [(6.11 ± 0.88) vs. (12.73 ± 0.26) pg/ml, *P* < 0.01], and higher levels of the anti-inflammatory cytokine IL-10 [(9.91 ± 0.42) vs. (5.26 ± 0.21) pg/ml, *P* = 0.012] than the control group (Table 2).

### Comparison of Organ Function, Treatment and Outcomes During Hospitalization Between the ACEI/ARB and Control Groups

Current users of ACEIs/ARBs seemed to have a higher rate of vasoconstrictive agent application (18 vs. 7%, *P* < 0.01) than the control group; however, the percentages of respiratory failure, shock, AKI, coagulation failure, and liver failure were not different between the two groups. In addition, the necessities for invasive IPPV and ECMO were not decreased in the ACEI/ARB group (Table 3).

**Table 3:** Organ function, treatments and outcomes in the two groups during hospitalization.

	**All (*n* = 152)**	**ACEI/ARB group (*n* = 38)**	**Control group (*n* = 114)**	***P***
Organ failure^*^, number (%)				
Respiratory failure	25 (16%)	8 (20%)	17 (15%)	0.092
Shock	13 (9%)	4 (11%)	8 (7%)	0.060
AKI	15 (10%)	4 (11%)	11 (10%)	0.829
Coagulation failure	3 (2%)	1 (3%)	2 (2%)	0.664
Liver failure	15 (10%)	4 (11%)	11 (10%)	0.796
**Treatment, number (%)**				
Antibiotics	105 (69%)	24 (64%)	81 (71%)	0.461
Antiviral treatment	145 (95%)	36 (92%)	109 (96%)	0.334
Glucocorticoids	49 (32%)	11 (30%)	38 (33%)	0.612
Intravenous immunoglobin	36 (24%)	9 (23%)	27 (24%)	0.552
Standard oxygen therapy	132 (87%)	35 (92%)	97 (85%)	0.080
HFNO	28 (18%)	7 (18%)	21 (18%)	0.927
NPPV	18 (12%)	5 (12%)	13 (11%)	0.327
IPPV	17 (11%)	4 (11%)	13 (11%)	0.629
ECMO	4 (3%)	1 (3%)	3 (3%)	0.994
Vasoconstrictive agents	15 (10%)	7 (18%)	8 (7%)	<0.01^b^
**Outcome**				
In-hospital progression^#^, number (%)	28 (18%)	6 (16%)	22 (19%)	0.326
In-hospital death, number (%)	15 (10%)	4 (10%)	11 (10%)	0.983
Hospital length of stay, days, mean ± SD	17 ± 8	24 ± 11	15 ± 7	<0.01^b^
Duration of viral shedding, days, mean ± SD	19 ± 3	24 ± 5	18 ± 5	0.034^a^
Time from onset to death or discharge, days, mean ± SD	27 ± 9	32 ± 10	25 ± 7	<0.01^b^

a *P < 0.05*;

b *P < 0.01*;

* *Shock was defined according to the interim guidance of the WHO for novel coronavirus (22, 23). AKI was identified and classified on the basis of the highest serum creatinine level or urine output criteria according to kidney disease, improving global outcome classification (23, 24). Respiratory failure, coagulation and liver failure were defined as a SOFA score greater than or equal to two points*.

# *Defined as a decline in PaO_2_/FiO_2_ > 100 mmHg or the need for IPPV and/or ECMO during hospitalization. AKI, acute kidney injury; HFNO, high flow nasal oxygenation; NPPV, noninvasive positive pressure ventilation; IPPV, invasive positive pressure ventilation; ECMO, extracorporeal membrane oxygenation*.

The duration of viral shedding [(24 ± 5) vs. (18 ± 5) days, *P* = 0.034], length of hospital stay [(24 ± 11) vs. (15 ± 7) days, *P* < 0.01], and time from onset to death or discharge [(32 ± 10) vs. (25 ± 7) days, *P* < 0.01] were longer in the ACEI/ARB group than in the control group, while no difference was found in the rate of in-hospital progression or death (Table 3).

### Subgroup Analyses: Comparison Between Patients Who Continued and Terminated Medication During Hospitalization

**Figure 2:**
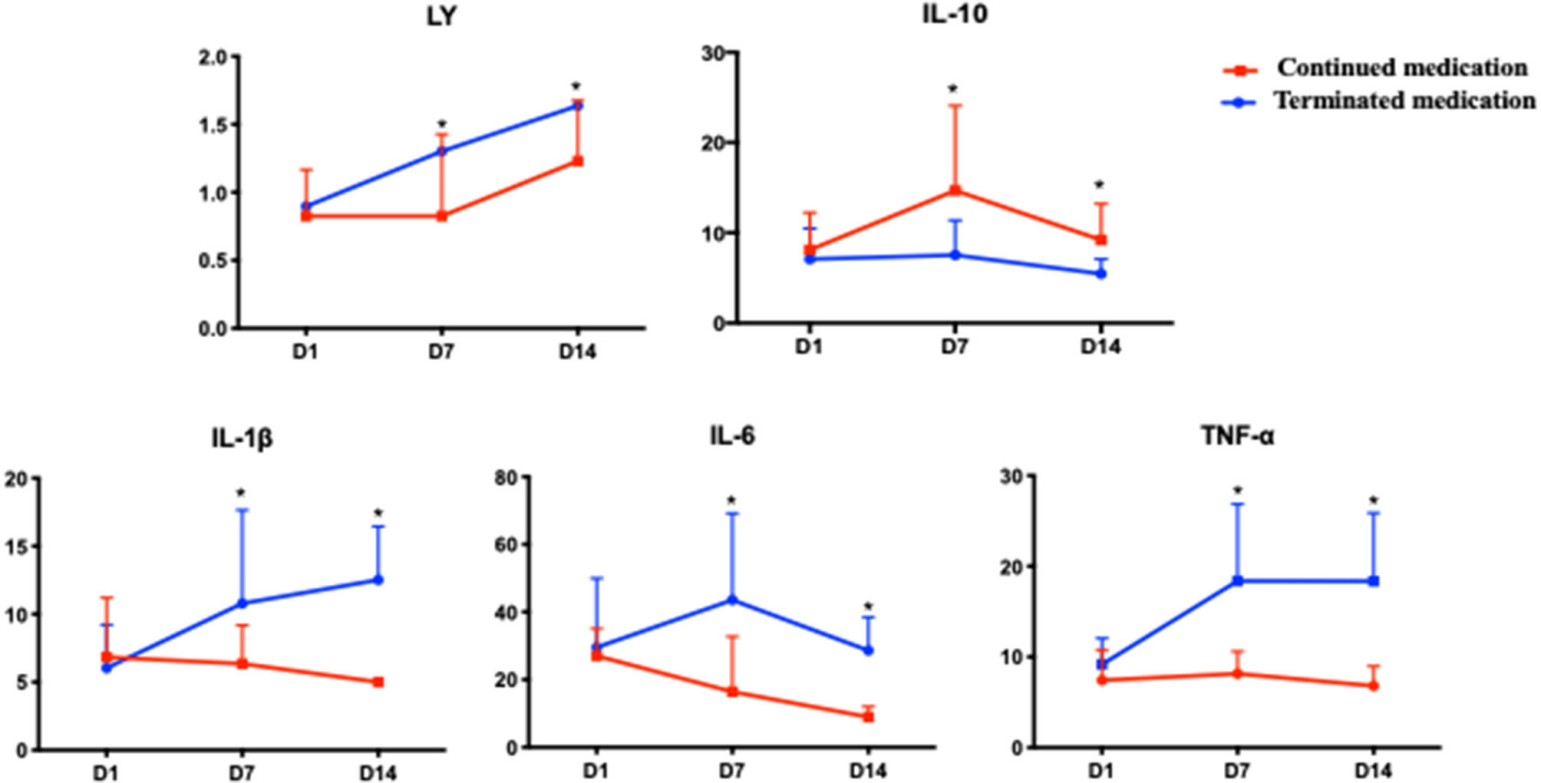
The dynamic changes in the lymphocyte counts and inflammatory factors between patients who continued and those who terminated ACEIs/ARBs during hospitalization. Patients with continued use of ACEIs/ARBs had consistently lower levels of lymphocytes, IL-1β, IL-6, and TNF-α but maintained higher levels of IL-10 on the seventh and fourteenth days than patients who terminated medication during hospitalization. However, the patients who terminated the medication had a trend of elevated lymphocyte counts and IL-1β from the first day to the fourteenth day. ^*^P < 0.01.

Among the patients in the ACEI/ARB group, 18 continued medication during hospitalization, while the other 20 terminated medication for several reasons. The baseline variables were with no significant difference between the two groups (Supplementary Table 1). The dynamic changes in lymphocytes and inflammatory factors at the first, seventh, and fourteenth days after hospitalization as well as the outcomes were compared between the two groups. The missing rates of IL-2R and IL-8 at seven days and 14 days after admission were extremely high and were not included in the analysis. Patients with continued use of ACEIs/ARBs had consistently lower levels of lymphocytes, IL-1β, IL-6, and TNF-α but maintained higher levels of IL-10 on the seventh and fourteenth days than patients who terminated medication during hospitalization. However, the patients who terminated the medication had a trend of elevated lymphocyte counts [day 1, day 7, day 14: (0.82 ± 0.47) vs. (1.41 ± 0.74) vs. (1.69 ± 0.45)^*^10^9^/L, *P* = 0.029] and IL-1β [day 1, day 7, day 14: (6.03 ± 3.19) vs. (10.78 ± 6.88) vs. (13.75 ± 5.26) pg/ml, *P* < 0.01] from the first day to the fourteenth day (Figure 2, Supplementary Table 2).

The duration of viral shedding [(27 ± 4) vs. (21 ± 5) days, *P* = 0.032], length of hospital stay [(26 ± 10) vs. (20 ± 3) days, *P* = 0.044], and time from onset to death or discharge [(34 ± 9) vs. (29 ± 10) days, *P* = 0.019] were longer in the continued medication group than in the terminated medication group. The rates of in-hospital progression and death were not significantly different between the two groups (Table 4).

**Table 4:** Outcomes in patients who continued and those who terminated ACEIs/ARBs during hospitalization.

**Outcomes**	**Continued ACEIs/ARBs** **(*n* = 18)**	**Terminated ACEIs/ARBs** **(*n* = 20)**	***P***
In-hospital progression^#^	3 (17%)	3 (15%)	0.611
In-hospital death	2 (11%)	2 (10%)	0.709
Duration of viral shedding, days	27 ± 4	20 ± 5	0.032^a^
Hospital length of stay, days	26 ± 10	20 ± 3	0.044^a^
Time from onset to death or discharge, days	34 ± 9	29 ± 10	0.019^a^

a *P < 0.05*;

# *Defined as a decline in PaO_2_/FiO_2_ > 100 mmHg or the need for IPPV and/or ECMO during hospitalization*.

### Subgroup Analyses: A Comparison of the Immune Status, Inflammatory Reactions and Outcomes Between the ACEI/ARB and Control Groups in Patients With Hypertension

Among 55 patients with hypertension, 30 patients were divided into the study group (ACEI/ARB group), and the other 25 patients were in the control group.

Compared with the control group, the patients in the study group had lower levels of IL-1β [(6.33 ± 0.56) vs. (8.27 ± 0.14) pg/ml, *P* = 0.026], IL-6 [(40.16 ± 12.59) vs. (52.33 ± 14.09) pg/ml, *P* = 0.030], and IL-8 [(31.60 ± 2.97) vs. (42.83 ± 3.27) pg/ml, *P* = 0.030] on admission. Regarding clinical outcomes, the duration of viral shedding [(26 ± 6) vs. (19 ± 4) days, *P* = 0.029] and time from onset to death or discharge [(30 ± 10) vs. (24 ± 8) days, *P* = 0.031] were longer in the study group than in the control group; however, no difference was detected in the rate of in-hospital progression and death between the two groups.

## Discussion

To our knowledge, this is the first study to thoroughly evaluate the inflammatory responses and viral clearance of COVID-19 patients treated with ACEIs/ARBs by a multicenter, retrospective cohort control study and to allow dynamic observation of inflammatory responses by continuous monitoring from the first to the fourteenth day after admission.

The major findings of our study were that ACEIs/ARBs inhibited the proinflammatory response but promoted the anti-inflammatory response and persistently decreased lymphocytes, thus extending the duration of viral shedding and the length of hospital stay. Antiviral treatments should be enforced in those patients. In addition, since current users of ACEIs/ARBs seem to have a higher necessity of vasoconstrictive agents, hemodynamics should be monitored closely during medication use. The message to the physician was that the influence on the ACEI/ARB treatment was limited, and they should not be withdrawn if there was no formal contraindication.

Inflammation is mediated by proinflammatory cytokines and anti-inflammatory cytokines. Inappropriate elevated expression of proinflammatory cytokines can result in sepsis, tissue destruction, or death (21, 24). Our study revealed that the plasma levels of IL-1β, IL-6, IL-8, and TNF-α in patients taking ACEI/ARBs were lower than those in patients not without medication; in addition, persistently lower levels of proinflammatory factors were maintained in patients who continued medication during hospitalization, which was consistent with the previous experimental results by Gullestad et al. (25) with the conclusion that high-dose enalapril was associated with a significant decrease in IL-6 activity in patients with severe chronic heart failure. The specific organ and systemic inflammatory responses were postulated to attenuate through a reduction in the level of cytokines, which might be explained by the attenuating effects of ACE inhibitors through the deactivation of the ACE-AngII-AT1 axis but the stimulation of the ACE2-Ang-(1-7)-Mas axis in a feedback mechanism (9, 26, 27) as a negative regulator with attenuated cytokines and thus protecting the patients from organ injury. Consequently, some authors (28, 29) have speculated that the use of ACEIs/ARBs might actually be a potentially beneficial intervention in those with COVID-19.

Apart from organ protection by attenuating the inflammatory response, basic investigation has shown that bradykinin and substance P produced by ACE inhibitors sensitize the sensory nerves of the airways and enhance the cough reflex (30, 31), which plays a protective role against pathogens. These two mechanics made it possible to improve the outcome in patients with pneumonia. Mortensen et al. (6) found a significant decrease in mortality, the length of hospital stay, and mechanical ventilation in patients taking ACE/ARBs who were hospitalized with pneumonia compared to a matched cohort. A meta-analysis (32) that included 19 studies noted that patients taking ACE inhibitors were associated with a significant approximately one-third reduction in the risk of pneumonia compared with controls. In addition, a recent study (8) by Christopher Henry also observed lower rates of death and intubation with continued use of ACE inhibitors than with terminated use (OR = 0.25; 95% CI, 0.09–0.64) throughout the hospital stay in cases of viral pneumonia not due to coronavirus. Unfortunately, our study did not find decreased mortality in patients with current use of ACEI/ARBs, even though we analyzed patients with continued medication during hospitalization and combined with hypertension to avoid potential confounding factors. The most likely explanation was that our study included a small number of patients, while most of their patients had mild cases as determined by SOFA scores and without excessive inflammatory reactions, which was the target for ACE inhibitors or ARBs.

What noteworthy was that ACEI/ARBs increased the necessity of vasoconstrictive agents. It could be explained by the nature of the antihypertensive agents and came as a revelation to us that the hemodynamics should be monitored closely during medication.

Our research also revealed that ACE inhibitors or ARBs led to prolonged viral shedding and extended the length of hospitalization. SARS-CoV-2 appears to bind to its target cells through angiotensin-converting enzyme 2 (ACE2). ACE inhibitors or ARBs upregulate ACE2 receptor expression in humans (33) by blocking the classic ACE pathway; thus, it is theoretically possible that the pre-existing use of these drugs might predispose a person to infection with a greater viral load of SARS-CoV-2 (13). This hypothesis was supported by the evidence of Ferrario that there was a 4.7-fold increase in cardiac ACE2 mRNA by an ACE inhibitor (34). Decreased lymphocyte counts and elevated plasma levels of IL-10 were also important discoveries in patients with ACEI/ARBs. Moreover, the lymphocyte counts in patients with continued use of medication during hospitalization recovered slowly, as observed by successive monitoring on the first to fourteenth days. The immune status was weakened by lymphocytopenia and elevated anti-inflammatory cytokines in patients taking ACEI/ARBs, which might be another reason for the slow viral clearance. As the important criterion for discharge was the negative conversion of the SARS-CoV-2, prolonged viral shedding led to an extended length of hospitalization. This might be the defect of the ACEI/ARBs and might explain the mixed results and controversy about their prescription in COVID-19 patients. For this reason, antiviral therapy in patients taking ACEI/ARBs should be reinforced, and their viral load should be monitored closely.

An autopsy report revealed that mononuclear inflammatory infiltration dominated by lymphocytes was observed in the lungs, but no virus inclusion bodies were found (35). We could then propose a hypothesis that cytokines released by inflammatory storms secondary to viral infection might be more important in the death of critically ill patients with COVID-19 than the viral infection itself in a certain period. From this perspective, it is possible that ACEI/ARBs might improve the outcome in critically ill patients with excessive inflammatory responses or severe multiple organ failure; when the inflammatory storm gradually diminishes, the focus of therapy should be on clearance of the virus and the enhancement of the immune system. Prospective cohort and randomized controlled trials are needed to confirm this hypothesis and examine potential mechanisms of action.

Our study was limited by the small number of patients included and by not strictly excluding confounding factors. We especially noticed that the number of patients with hypertension was much higher in the ACEI/ARB group, which might be an important confounding factor. However, by subgroup analyze in patients with hypertension, we found similar results. The prospective randomized controlled studies designed by increasing the sample size and strictly excluding potential confounders to explore the impact of ACE/ARBs on inflammatory responses, viral clearance and the mortality in COVID-19 patients should be encouraged in the future.

## Data Availability Statement

The original contributions presented in the study are included in the article/Supplementary Materials, further inquiries can be directed to the corresponding author.

## Ethics Statement

The studies involving human participants were reviewed and approved by the Ethics Committee of China-Japan Friendship Hospital approved this study. Written informed consent for participation was not required for this study in accordance with the national legislation and the institutional requirements.

## Author Contributions

All authors made substantial contributions to the conception and design of the study or to the data acquisition, analysis, or interpretation, reviewed and approved the final manuscript, and significantly contributed to this study. QZ took full responsibility for the integrity of the submission and publication and was involved in the study design. LHuang and ZC involved in data collection, had full access to all of the data in the study, took responsibility for the integrity of the data and were responsible for data verification, as well as the drafting of the manuscript. LHua took the responsibility for statistical analysis and the accuracy of the data analysis. Others involved in data collection, had full access to all of the data in the study, and took responsibility for the integrity of the data.

## Conflict of Interest

The authors declare that the research was conducted in the absence of any commercial or financial relationships that could be construed as a potential conflict of interest.

## Publisher's Note

All claims expressed in this article are solely those of the authors and do not necessarily represent those of their affiliated organizations, or those of the publisher, the editors and the reviewers. Any product that may be evaluated in this article, or claim that may be made by its manufacturer, is not guaranteed or endorsed by the publisher.
