# Supplementary material for: Impact of Angiotensin-Converting Enzyme Inhibitors and Angiotensin Receptor Blockers on the Inflammatory Response and Viral Clearance in COVID-19 Patients
Source: Front Cardiovasc Med. 2021 Aug 19;8:710946. doi: 10.3389/fcvm.2021.710946 (PMC8416906; doi:10.3389/fcvm.2021.710946)
Supplement: Supplementary file 1 [file Table_1.docx]

**Supplement 1. Baseline variables in patients who**

**continued and terminated medication during hospitalization**

|  | **Continued**  **ACEIs/ARBs**  **(n=18)** | **Terminated**  **ACEIs/ARBs**  **(n=20)** | ***P*** |
| --- | --- | --- | --- |
| **Age, years, mean ± SD** | 57±12 | 57±11 | 0.926 |
| **Gender (men), number (%)** | 9 (50%) | 10 (50%) | 1 |
| **Body mass index, kg/m^2^, mean ± SD** | 21.2±6.1 | 21.0±7.2 | 0.911 |
| **History of smoking, number (%)** | 4 (22%) | 4 (20%) | 0.605 |
| **Other medication history within 90 days, number (%)** |  |  |  |
| Corticosteroids | 0 (0%) | 0 (0%) | 1 |
| Immunosuppressants | 0 (0%) | 0 (0%) | 1 |
| Statins | 3 (14%) | 3 (15%) | 0.639 |
| Thiazolidinediones | 0 (0%) | 0 (0%) | 1 |
| α receptor blocking agent | 1 (1%) | 0 (0%) | 0.887 |
| β receptor blocking agent | 2 (11%) | 3 (15%) | 0.201 |
| CCB | 2 (11%) | 3 (15%) | 0.201 |
| Diuretics | 2 (11%) | 2 (10%) | 0.762 |
| **SOFA Score, points (IQR)** | 1.5 (1-2) | 1.5 (1-3) | 0.972 |
| **CCI, points (IQR)** | 1 (1-2) | 1 (1-2) | 1 |
| **Treatment before hospital, number (%)** |  |  |  |
| Methylprednisolone | 1 (6%) | 2 (10%) | 0.081 |
| Antibiotic therapy | 10 (56%) | 12 (60%) | 0.403 |
| Antiviral therapy | 11 (61%) | 11 (55%) | 0.082 |

CCB, calcium channel blocker; SOFA, Sequential Organ Failure Assessment; CCI, Charlson’s Comorbidity Index
