# Supplementary material for: Impact of Angiotensin-Converting Enzyme Inhibitors and Angiotensin Receptor Blockers on the Inflammatory Response and Viral Clearance in COVID-19 Patients
Source: Front Cardiovasc Med. 2021 Aug 19;8:710946. doi: 10.3389/fcvm.2021.710946 (PMC8416906; doi:10.3389/fcvm.2021.710946)
Supplement: Supplementary file 2 [file Table_2.docx]

**Supplement 2. The dynamic changes in the lymphocytes and inflammatory factors**

**in patients continued and terminated ACEI/ARB during hospitalization**

|  | **D1** | **D7** | **D14** | ***P*** |
| --- | --- | --- | --- | --- |
| **Continued medication group,** **mean ± SD** |  |  |  |  |
| Lymphocytes, *10^9/L | 0.79±0.52 | 0.80±0.23 | 1.23±0.33 | 0.074 |
| IL-1β, pg/mL | 6.85±4.37 | 6.15±1.77 | 4.86±0.24 | 0.153 |
| IL-6, pg/mL | 32.17±4.33 | 19.43±6.90 | 10.22±2.14 | 0.070 |
| IL-10, pg/mL | 9.21±4.14 | 14.69±9.41 | 13.82±5.02 | 0.095 |
| TNF-α, pg/mL | 7.85±2.83 | 8.17±2.45 | 7.81±2.17 | 0.639 |
| **Terminated medication group,**  **mean ± SD** |  |  |  |  |
| Lymphocyte count, *10^9/L | 0.82±0.47 | 1.41±0.74 | 1.69±0.45 | 0.029 ^a^ |
| IL-1β, pg/mL | 6.03±3.19 | 10.78±6.88 | 13.75±5.26 | 0.002 ^b^ |
| IL-6, pg/mL | 32.76±15.92 | 41.44±20.32 | 33.20±7.29 | 0.098 |
| IL-10, pg/mL | 8.08±3.40 | 7.71±3.94 | 5.53±1.52 | 0.090 |
| TNF-α, pg/mL | 9.19±2.86 | 19.11±6.21 | 20.03±4.26 | 0.067 |

^b^: *P*<0.01; IL-1β, interleukin 1β; TNF-α, tumor necrosis factor α; D1, the first day after admission; D7, the seventh day after admission; D14, the fourteenth day after admission
